# AMPK contributes to autophagosome maturation and lysosomal fusion

**DOI:** 10.1038/s41598-018-30977-7

**Published:** 2018-08-23

**Authors:** Minsu Jang, Rackhyun Park, Hyunju Kim, Sim Namkoong, Daum Jo, Yang Hoon Huh, Ik-Soon Jang, Jin I. Lee, Junsoo Park

**Affiliations:** 1 Division of Biological Science and Technology, Yonsei University, Wonju, Republic of Korea; 2 Center for Electron Microscopy Research, Korea Basic Science Institute, Ochang-eup, Cheongju-si Republic of Korea; 3 Division of Bioconvergence Analysis, Korea Basic Science Institute, Daejeon, Republic of Korea

## Abstract

AMP-activated protein kinase (AMPK) regulates autophagy initiation when intracellular ATP level decreases. However, the role of AMPK during autophagosome maturation is not fully understood. Here, we report that AMPK contributes to efficient autophagosome maturation and lysosomal fusion. Using CRISPR-Cas9 gene editing, we generated AMPK α1 knockout HEK293T cell lines, in which starvation-induced autophagy is impaired. Compound C, an AMPK-independent autophagy inducer, and trehalose, an mTOR-independent autophagy inducer were used to examine the role of AMPK in autophagosome maturation and lysosomal fusion. While the treatment of control cells with either compound C or trehalose induces activation of autophagosomes as well as autolysosomes, the treatment of AMPK α1 knockout cells with compound C or trehalose induces mainly activation of autophagosomes, but not autolysosomes. We demonstrate that this effect is due to interference with the fusion of autophagosomes with lysosomes in AMPK α1 knockout cells. The transient expression of AMPK α1 can rescue autophagosome maturation. These results indicate that AMPK α1 is required for efficient autophagosome maturation and lysosomal fusion.

## Introduction

Autophagic flux is the entire process of macroautophagy (hereafter referred to as autophagy), ranging from the inclusion of cargo within the autophagosome to digestion in the autolysosome, and either increased autophagic flux or a block in autophagic flux can result in autophagosome accumulation^1^. During the process of increased autophagic flux, the autophagosome fuses with the lysosome to form an autolysosome, which provides an acidic environment for lysosomal hydrolases to destroy the cargo molecules^2,3^. Autophagosome maturation and the lysosomal fusion process can be analyzed by tandem fluorescent-tagged LC3 (ptf-LC3) or the level of p62/SQSTM1^2,4,5^.

AMP activated protein kinase (AMPK) is a crucial cellular energy sensor protein and is activated by a low energy state in the cell^6,7^. The AMPK complex consists of catalytic α subunits and regulatory β and γ subunits, and the mammalian genome has multiple AMPK subunit isoforms (α1, α2, β1, β2, γ1, γ2, γ3)^8^. The expression of AMPK α1 complex is ubiquitous; however, the expression of AMPK α2 is high in skeletal muscle, the heart, and the liver^9,10^. AMPK is one of the major autophagy regulators, and the role of AMPK in autophagy initiation has been clearly shown. Under glucose starvation, AMPK associates with and activates autophagy-initiating kinase Ulk1, which is an orthologue of yeast ATG1, the most upstream component of the autophagy machinery^11–13^. In addition, the activation of AMPK can phosphorylate TSC2 and the activated TSC2 can suppress mTOR complex 1 (mTORC1) to induce autophagy^14,15^. However, the role of AMPK in autophagosome maturation and lysosome fusion is not fully understood. Several reports have suggested that AMPK is involved in autophagosome maturation. Although AMPK can negatively regulate mTORC1 signaling and mTORC1 activation can suppress autophagosome maturation via UVRAG phosphorylation^16,17^, the relationship between AMPK and activation of autophagosome maturation is not clear. Metformin, an activator of AMPK, can induce autophagy, as can compound C, an inhibitor of AMPK^18–20^. Compound C induced autophagosome formation in an AMPK-independent manner, since neither the AMPK activator, AICAR nor metformin blocked compound C-induced autophagosome formation^19^.

Trehalose, a disaccharide present in non-mammalian species, inhibits solute carrier 2 A (SLC2A) and induces an mTOR independent autophagy^21–23^.

In this report, we generated AMPK α1 knockout cell lines, which impaired starvation-induced autophagy. Because the transfection efficiency of HEK293T cells is high, knockout HEK293T cells were used for transient expression experiments involving the autophagy marker and cell signaling reporter. Compound C and trehalose treatment induced autophagosome formation in both control and AMPK α1 knockout cells. However, autophagosome maturation and lysosome fusion were blocked in AMPK α1 knockout cells. The overexpression of AMPK rescued AMPK function, indicating that AMPK is required for efficient autophagic flux even though compound C-induced autophagosome formation is AMPK independent.

## Results

### Generation of AMPK α1 knockout (KO) HEK293T cells

**Figure 1:**
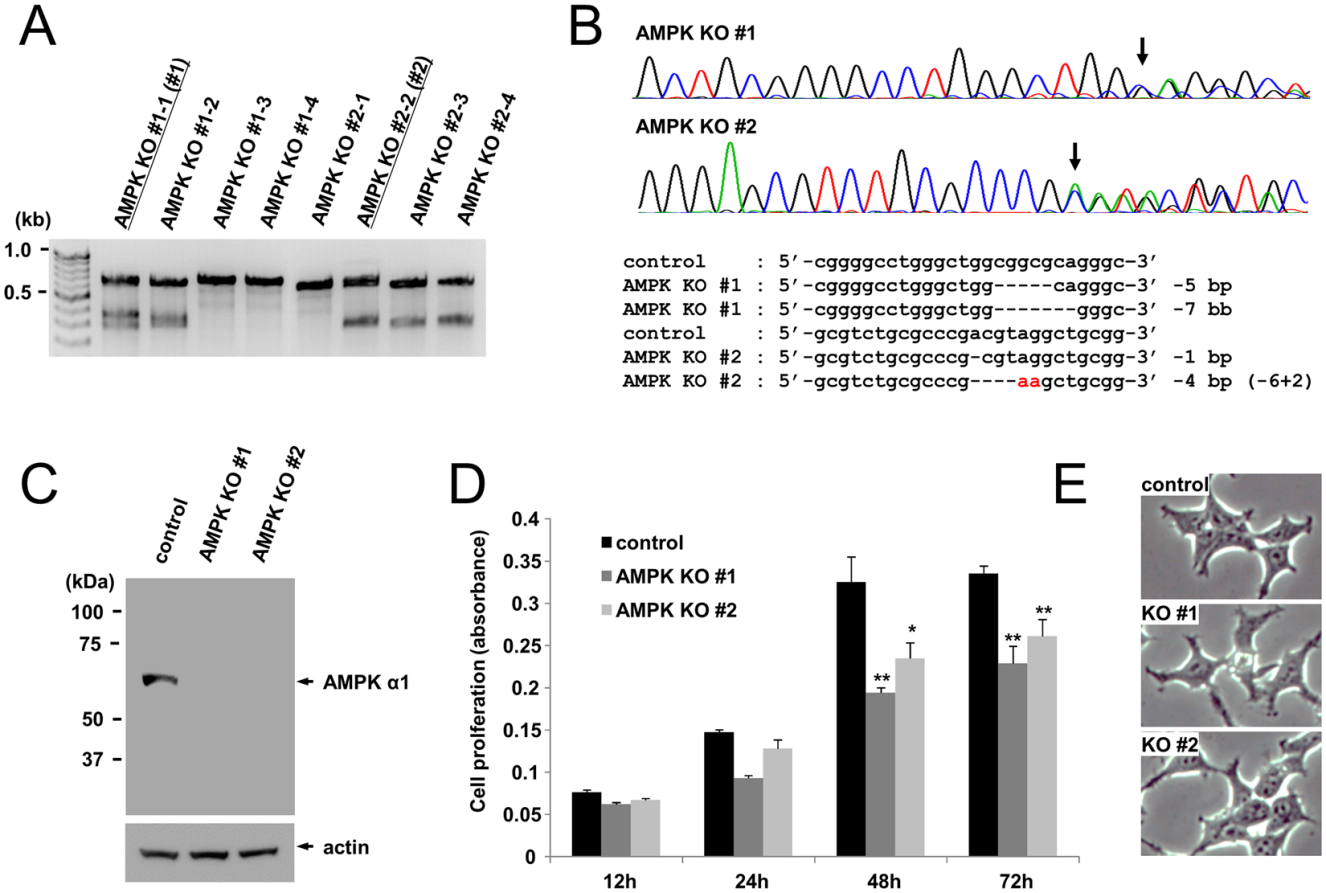
Generation of AMPK α1 knockout (KO) HEK293T cells. (**A**) Validation of AMPK α1 KO by T7 endonuclease 1 (T7E1) assay. HEK293T cells were transfected with either pX459/AMPK α1 gRNA #1 or pX459/AMPK α1 gRNA #2, and single colonies were isolated. The genomic PCR products were analyzed by T7E1 assay. Two different clones (KO #1 and KO #2) were used for the further experiments. (**B**) DNA sequencing analysis revealed the presence of AMPK α1 mutation in KO #1 and KO #2 (Top). The CRISPR-Cas9 system introduced an indel mutation in the target sites of the AMPK α1 gene (bottom). (**C**) Validation of AMPK α1 KO by Western blotting. Equal amounts of HEK293T wild-type (WT) and AMPK α1 KO cell lysates were subjected to Western blotting with an anti-AMPK α1 antibody. Experiments were repeated three times with similar results. (**D**) Proliferation of AMPK α1 KO cell lines was analyzed by MTT assay. The MTT assay was performed in triplicate, and the graph shows the average and standard deviation (SD). Control vs. knockout cells. *P < 0.05, **P < 0.01. (**E**) Phase contrast microscope images (400x) of control cells and AMPK α1 knockout cells.

We generated AMPK α1 knockout (KO) cell lines using the CRISPR-Cas9 gene editing system^24^. Two AMPK α1 guide RNA sets were synthesized and cloned into a pX459 vector. AMPK α1 knockout plasmids were transfected into HEK293T cells. After selection, we isolated single colonies and analyzed the insertion or deletion mutation (indel) using T7 endonuclease 1 (T7E1) assays (Fig. 1A). Next, we analyzed the indel mutation of the PCR products of target DNA by nucleotide sequencing and confirmed that the AMPK α1 gene was mutated (Fig. 1B). Finally, we demonstrated that the expression of AMPK α1 protein was abolished in HEK293T cells by Western blotting (Fig. 1C). These results collectively indicate that AMPK α1 knockout cell lines were successfully established by the CRISPR-Cas9 system. Because gene knockout often affects cell proliferation, we examined the cell proliferation of AMPK α1 knockout cells by MTT assay. Although there was no remarkable phenotypic change, the proliferation of AMPK α1 knockout cells was significantly reduced by up to 25% compared to HEK293T control cells (Fig. 1D,E).

### AMPK α1 knockout impairs starvation-induced autophagy

**Figure 2:**
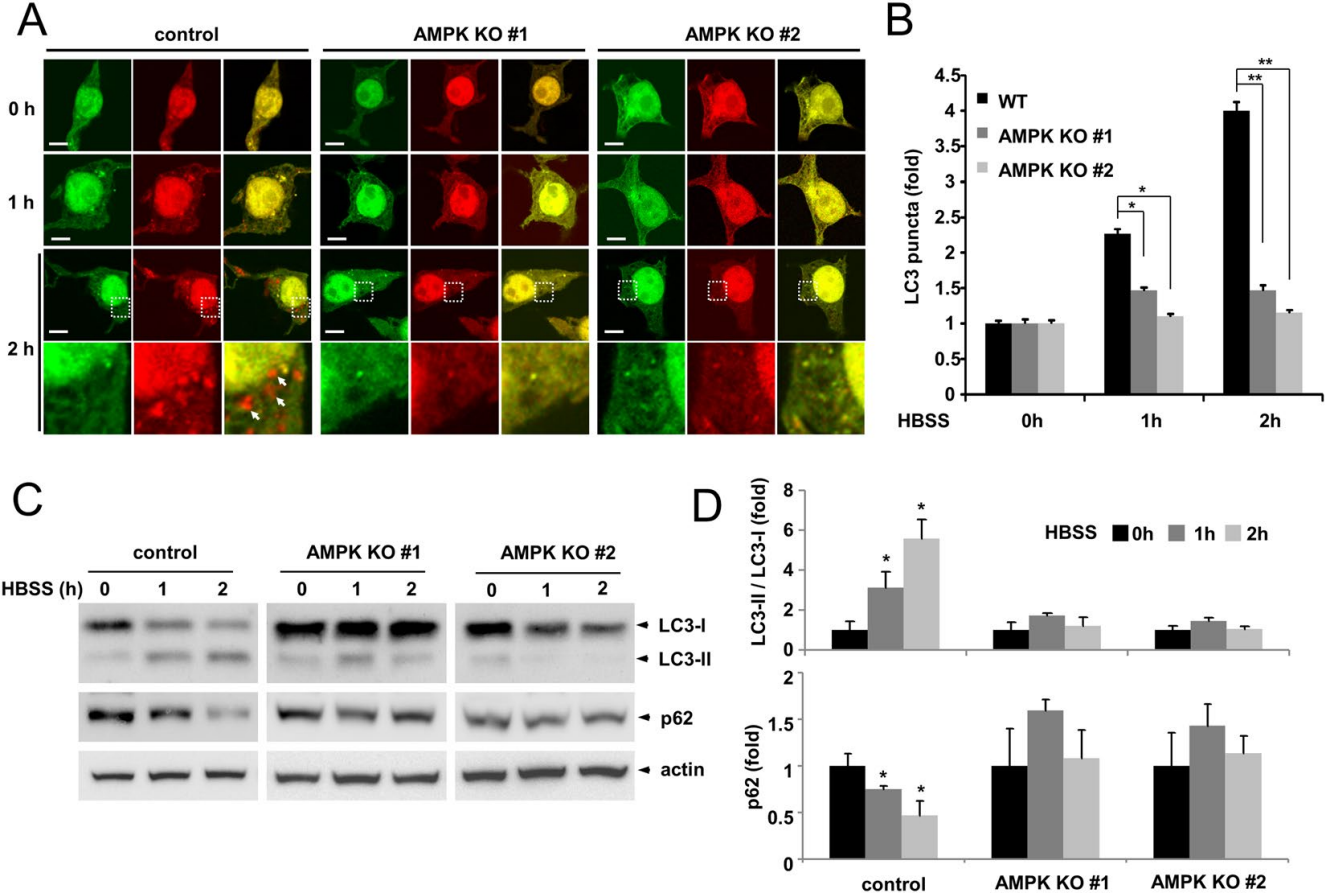
AMPK α1 knockout inhibits autophagy initiation upon starvation. (**A**) Control cells and AMPK α1 knockout cells were transfected with a plasmid encoding mRFP-GFP-LC3, and cells were either mock-treated or starved in HBSS medium for the indicated time (1 h or 2 h). Bars: 10 μm. (**B**) The numbers of LC3 puncta in control cells and AMPK α1 knockout cells were counted (n = 20). Control cells vs. AMPK knockout cells, *P < 0.05, **P < 0.0005. (**C**) AMPK α1 knockout represses starvation-induced autophagy. Control cells and AMPK α1 knockout cells were incubated in HBSS medium, and the cell lysates were probed with anti-LC3 and anti-p62 antibodies. (**D**) The LC3-II, LC3-I and p62 bands were quantified, and the relative expression levels of LC3-II/LC3-I and p62 are shown in the graph. 0 h vs. 1 h or 2 h, *P < 0.05.

Many reports support that AMPK is required for autophagy initiation upon cellular starvation^12,25^. We examined whether AMPK α1 is required for autophagy using AMPK α1 knockout cells. To examine the regulation of autophagy by AMPK, we used an mRFP-GFP-LC3 reporter construct (ptfLC3), which appears yellow when autophagosomes form and red when autolysosomes form^2^. Control cells and AMPK α1 knockout cells were incubated with HBSS for 1 h and 2 h, respectively, and cells were observed under a fluorescence microscope. HEK293T control cells showed an increased number of yellow and red puncta (autophagosome/autolysosome), However, AMPK α1 knockout cells showed only a limited number of yellow puncta, indicating that autophagy initiation was impaired (Fig. 2A,B). We also examined the autophagy markers LC3 and p62. In control cells, the level of LC-II/LC3-I protein was gradually elevated, and the level of p62 was reduced, however, AMPK α1 knockout cells did not show any significant changes (Fig. 2C,D). These results confirm that AMPK α1 is required for efficient autophagy initiation in HEK293T cells.

### Repression of autophagic flux in AMPK α1 knockout cells

**Figure 3:**
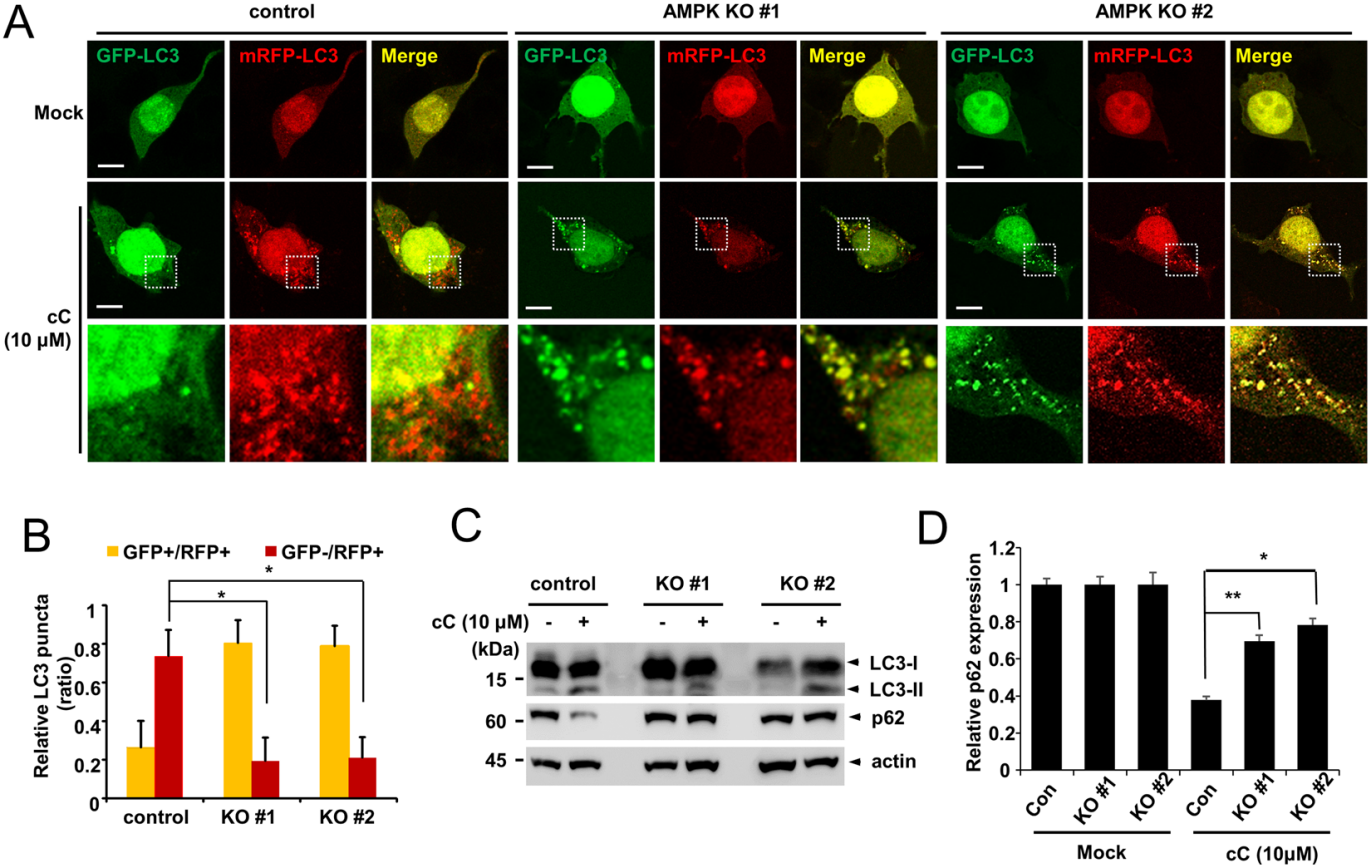
AMPK α1 knockout represses autophagic flux. (**A**) Control cells and AMPK α1 knockout cells were transfected with a plasmid encoding mRFP-GFP-LC3, and cells were either mock-treated (vehicle alone) or treated with compound C (cC, 10 μM) for 18 h. Bars: 10 μm. (**B**) Ratio quantification of autophagosomal LC3 puncta to autolysosomal LC3 puncta (n = 10). Control cells vs. knockout cells, *P < 0.0001. (**C**,**D**) HEK293T cells and AMPK α1 knockout cells were treated with compound C (10 μM) for 18 h, and the cell lysates were probed with anti-LC3 and anti-p62 antibodies. The p62 bands were quantified, and the relative expression levels are shown in the graph. Control cells vs. AMPK α1 knockout cells, *P < 0.05, **P < 0.005.

A previous study demonstrated that compound C, an inhibitor of AMPK, can induce autophagy in an AMPK-independent manner^19^. For this reason, we examined whether compound C could induce autophagy in AMPK α1 knockout cells. Control cells and AMPK α1 knockout cells were transfected with an mRFP-GFP-LC3 reporter construct (ptfLC3), and cells were treated with compound C for 18 h. Both control cells and AMPK α1 knockout cells showed cytoplasmic puncta, suggesting that autophagosomes had formed. While control cells showed red puncta (autolysosomes), AMPK α1 knockout cells showed yellow puncta (autophagosomes), indicating that autophagic flux was impaired in AMPK α1 knockout cells (Fig. 3A,B). We also examined the expression levels of autophagy markers LC3 and p62. While the level of p62 protein in HEK293T control cells was decreased with compound C treatment, this decrease was less prominent in AMPK α1 knockout cells (Fig. 3C,D). In addition, the treatment of a lysosomal inhibitor, bafilomycin A1 in combination with compound C showed that AMPK α1 knockout cells showed a decreased difference in p62 level between bafilomycin A1 treatment and untreated samples. This indicates a decreased autophagic flux in AMPK α1 knockout cells (Figs S1 and S2). Overall, these results indicate that autophagic flux in AMPK α1 knockout cells was impaired with compound C treatment. We also examined the level of LC3-II protein. Both HEK293T control cells and AMPK α1 knockout cells showed elevated levels of LC3-II, confirming that autophagosomes were formed in both cell lines (Fig. 3C). However, the level of LC3-II was not significantly changed in AMPK α1 knockout cells (Fig. S3). These results collectively indicate that AMPK α1 is not required for autophagosome formation after treatment with compound C, but is necessary for the successful formation of autolysosomes that occurs as a result of autophagic flux.

### Disruption of AMPK α1 interferes with the formation of autolysosomes

**Figure 4:**
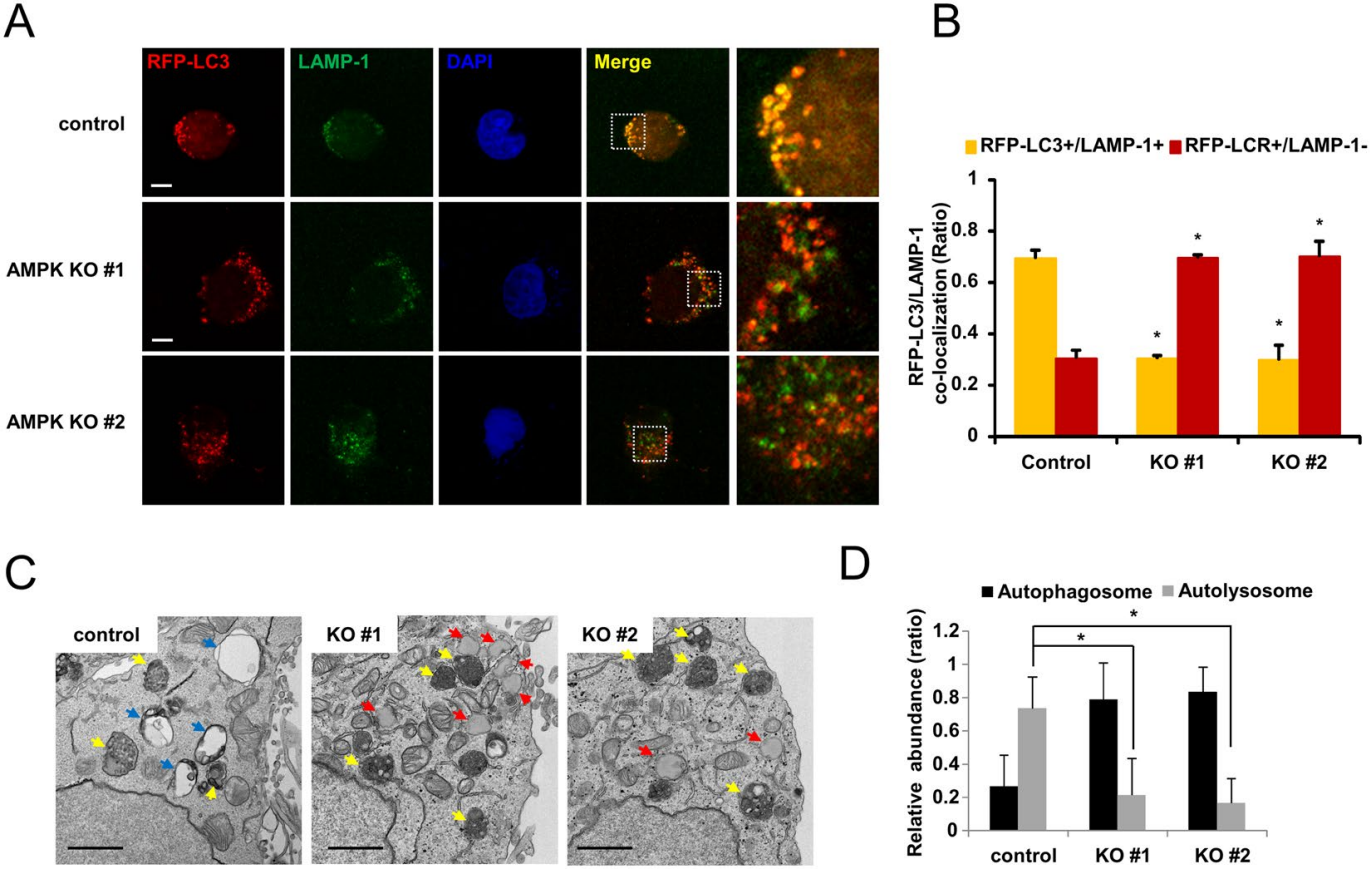
AMPK α1 knockout interferes with the formation of autolysosomes. (**A**) Control cells and AMPK α1 knockout cells were transfected with the plasmid encoding mRFP-LC3 and treated with compound C for 18 h. Cells were fixed and stained with an anti-LAMP1 antibody. Bars: 10 μm. (**B**) Ratio quantification of autophagosomal LC3 puncta (RFP-LC3 only) to autolysosomal LC3 puncta (RFP-LC3 and LAMP-1) Each data represents the mean and standard deviation of two independent experiments (n = 30), control cells vs AMPK Knockout cells, *P < 0.05. (**C**) Representative electron micrograph of HEK293T cells and AMPK α1 knockout cells after compound C treatment. Yellow arrows denote the autophagosome; blue arrows, autolysosomes; and red arrows, lysosomes. Bars: 1 μm. (**D**) Ratio quantification of autophagosomal puncta to autolysosomal puncta. We counted autophagosomes and autolysosomes in 54 individual cells (n = 54) of each group, and calculated the relative abundance. Graph shows the average and standard deviations. Control cells vs. knockout cells, *P < 0.0001.

To form mature autolysosomes, autophagosomes need to be fused with lysosomes. Because AMPK α1 knockout cells did not form autolysosomes with compound C treatment (Fig. 3A), we examined whether the autophagosomes could fuse with lysosomes in AMPK α1 knockout cells. Since GFP-LC3 protein loses its fluorescence in low lysosomal pH, we used mRFP-LC3 for the detection of autophagosomes and autolysosomes. Control cells and AMPK α1 knockout cells were transfected with mRFP-LC3. Twenty-four h after transfection, the cells were treated with compound C, followed by immunostaining with anti-LAMP1 antibody (a lysosomal marker). We found many mRFP-LC3 puncta co-stained with anti-LAMP1 in control cells; however, mRFP-LC3 puncta were positive for LAMP1 staining in only a few AMPK α1 knockout cells (Fig. 4A,B). These results suggest that the formation of mature autolysosomes was impaired in AMPK α1 knockout cells due to disruption of the fusion of autophagosomes with lysosomes. In order to confirm that AMPK α1 knockout interferes with autolysosome formation, we employed transmission electron microscopy to assess the ultra-structure of autophagosomes and autolysosomes. Control cells and AMPK α1 knockout cells were treated with compound C for 18 h, and then cells were fixed and examined with a transmission electron microscope. While the control cells showed autolysosomes as well as autophagosomes, the AMPK α1 knockout cells showed mainly autophagosomes and lysosomes (Fig. 4C,D). These results collectively indicate that AMPK is involved in autophagosome maturation and lysosome fusion.

### Disruption of AMPK α1 inhibits AMPK-related signaling

**Figure 5:**
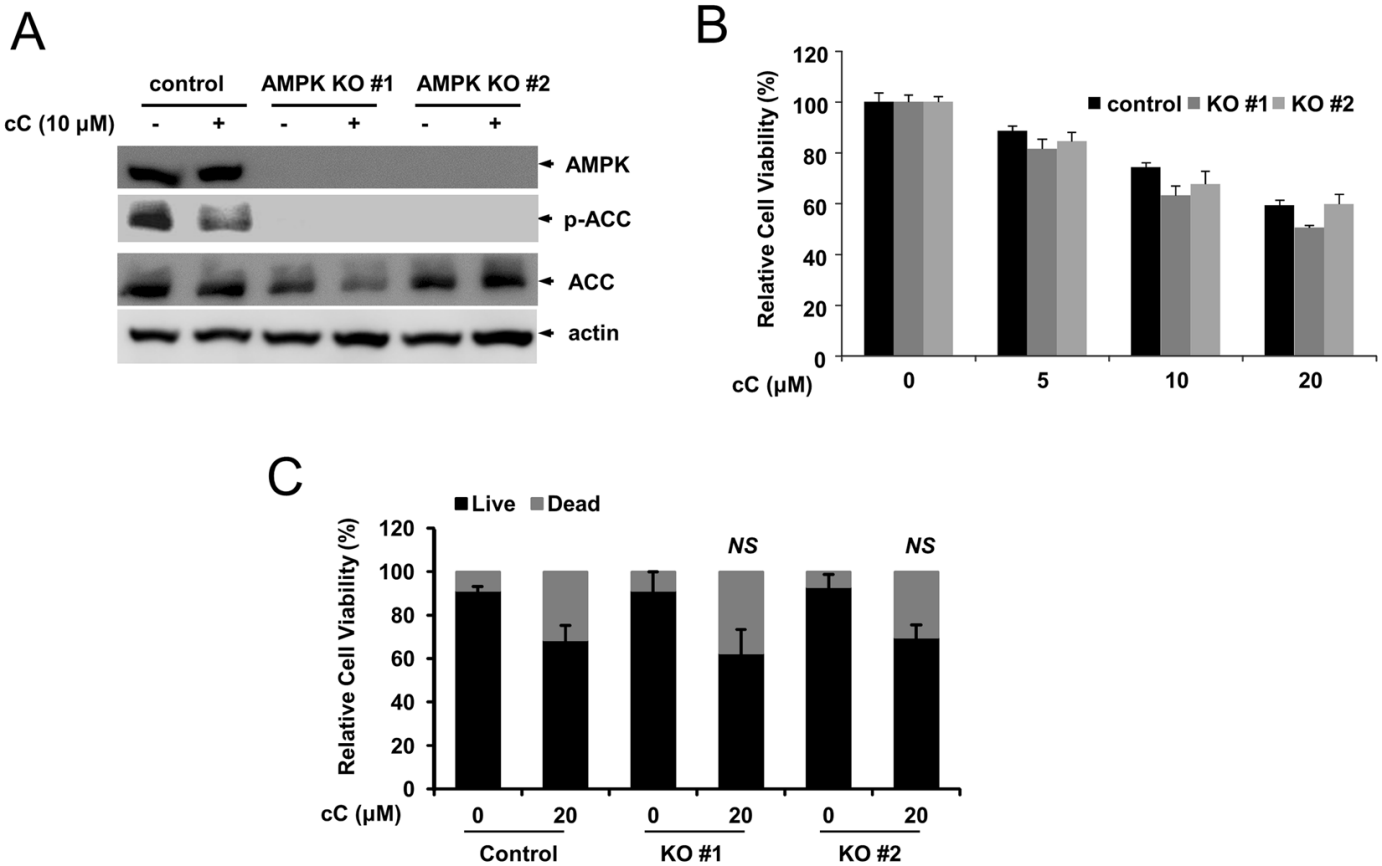
AMPK α1 knockout deregulates AMPK-related signaling. (**A**) AMPK α1 knockout decreases the phosphorylation level of acetyl-CoA carboxylase (ACC). Control cells and AMPK α1 knockout cells were either mock-treated (vehicle alone) or treated with compound C (cC, 10 μM) for 18 h, and the equal amount of cell lysates were probed with the indicated antibodies. (**B**) Proliferation of AMPK α1 KO cell lines after compound C treatment. HEK293T control and knockout clones were seeded into 24-well plates and incubated with the indicated concentrations of compound C. MTT assays were performed in triplicate, and the graph shows the average and standard deviation (SD). (**C**) Cell viability of AMPK α1 KO cell lines were measured with Trypan blue assay after compound C treatment. Control cells vs. knockout cells, NS: not significant.

We examined AMPK activity in AMPK α1 knockout cells that were treated with compound C, a selective AMPK inhibitor. AMPK is the main regulator of acetyl-CoA carboxylase (ACC), which can be phosphorylated by AMPK activation^26^. Control cells and AMPK α1 knockout cells were either mock treated or treated with compound C, and the cell lysates were probed with an anti-phospho-ACC antibody (p-ACC). While the level of phospho-ACC was decreased in control cells with compound C treatment, phospho-ACC was hardly detectable in AMPK α1 knockout cells (Fig. 5A). These results demonstrate that AMPK α1 is required for the phosphorylation of ACC. In addition, we examined cell viability upon treatment with compound C. Cell viability was decreased by compound C in a dose-dependent manner, However, the cell viability of AMPK knockout cells was not clearly different from that of the control cells (Fig. 5B,C).

### Transient expression of AMPK α1 rescues the AMPK α1 knockout phenotype

**Figure 6:**
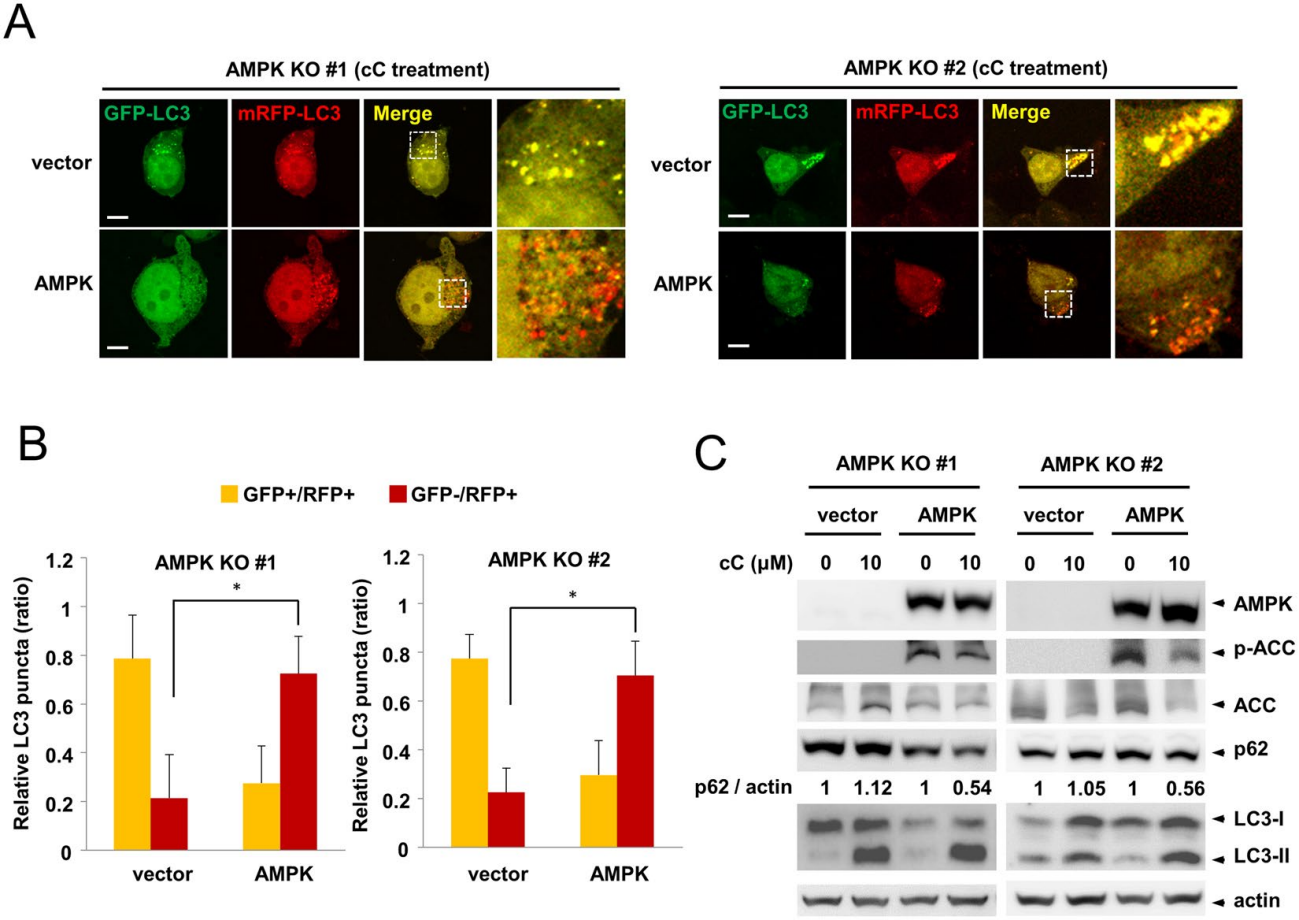
Expression of AMPK α1 recues the AMPK α1 knockout phenotype. (**A**) AMPK α1 knockout cells were transfected with mRFP-GRP-LC3 in the presence or absence of the AMPK plasmid. Twenty-four h after transfection, cells were treated with compound C for 18 h. Bars: 10 μm. (**B**) Ratio quantification of autophagosomal LC3 puncta to autolysosomal LC3 puncta (n = 10). Vector vs AMPK α1 plasmid, *P < 0.0001. (**C**) AMPK α1 knockout cells were transfected with the plasmid encoding AMPK α1. Twenty-four hour after transfection, cells were treated with compound C for 18 h. Equal amounts of cell lysates were probed with the indicated antibodies.

Because AMPK α1 knockout cells showed impairment in autophagosome maturation, we examined whether the overexpression of AMPK α1 can rescue AMPK activity and autophagosome maturation. AMPK α1 knockout cells were transfected with an AMPK α1 plasmid encoding full-length AMPK α1 cDNA and an mRFP-GFP-LC3 plasmid, and the cells were then treated with compound C. Transient expression of AMPK α1 resulted in the presence of autolysosome puncta (red), indicating efficient autophagic flux (Fig. 6A,B). In addition, the restoration of AMPK α1 resulted in the phosphorylation of ACC (Fig. 6C). Finally, expression of AMPK α1 resulted in a reduction in p62 level after compound C treatment (Fig. 6C). These results collectively indicate that AMPK α1 expression can rescue the AMPK α1 knockout phenotype.

### AMPK α1 knockout represses trehalose induced autophagic flux

**Figure 7:**
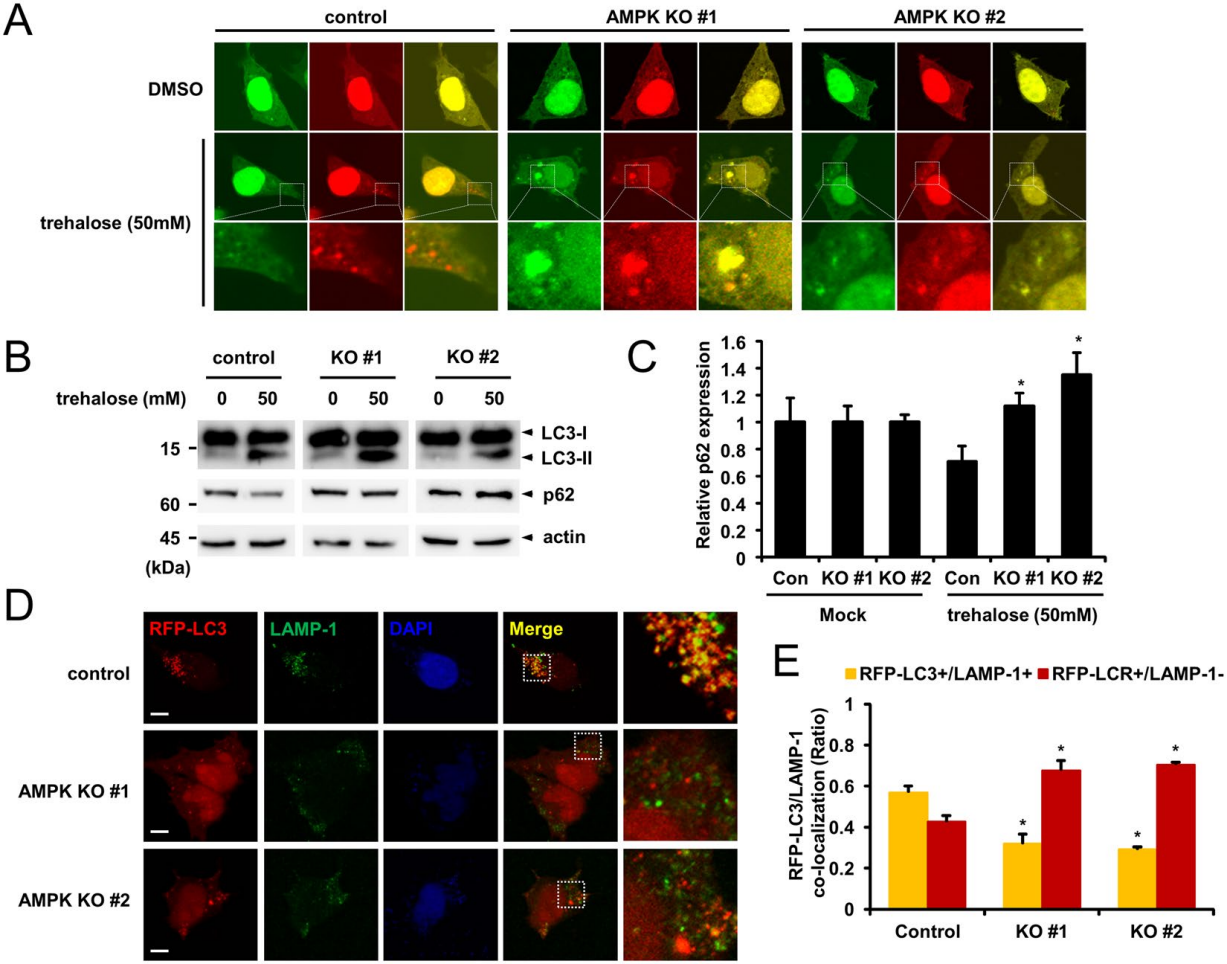
AMPK α1 knockout represses trehalose induced autophagic flux. (**A**) Control cells and AMPK α1 knockout cells were transfected with mRFP-GRP-LC3. Twenty-four h after transfection, cells were either mock-treated (vehicle only) or treated with trehalose (50 mM) for 4 h. (**B**,**C**) HEK293T cells and AMPK α1 knockout cells were treated with trehalose (50 mM) for 6 h, and the cell lysates were probed with anti-LC3 and anti-p62 antibodies. The p62 bands were quantified, and the relative expression levels are shown in the graph. Control cells vs. AMPK α1 knockout cells, *P < 0.05. (**D**,**E**) Cells were transfected with mRFP-LC3 plasmid. 24 h after, cells were treated with Trehalose for 6 h. Cells were fixed and stained with anti-LAMP1 antibody. Ratio quantification of autophagosomal LC3 puncta (RFP-LC3 only) to autolysosomal LC3 puncta (RFP-LC3 and LAMP-1). Each data represents the mean and standard deviation of two independent experiments (n = 30), control cells vs AMPK Knockout cells, *P < 0.05.

Since the AMPK inhibitor compound C induces autophagy independent of AMPK, yet regulates autophagic flux AMPK-dependently, we sought to clarify the role of AMPK in autophagic flux by inducing autophagy in another manner. A previous study showed that trehalose can induce autophagy in mTOR independent manner^21^. Control cells and AMPK α1 knockout cells were transfected with an mRFP-GFP-LC3 reporter construct (ptfLC3), and cells were treated with trehalose. Both control cells and AMPK α1 knockout cells showed cytoplasmic puncta, suggesting that autophagosomes had formed. While control cells showed mainly red puncta (autolysosomes), AMPK α1 knockout cells showed yellow puncta (autophagosomes) (Fig. 7A). We also examined the expression levels of autophagy markers LC3 and p62. While the level of p62 protein in control cells was decreased with trehalose treatment, the level of p62 protein was significantly increased in AMPK α1 knockout cells (Fig. 7B,C). The level of LC3-II was not significantly changed in AMPK α1 knockout cells (Fig. S4).

**Figure 8:**
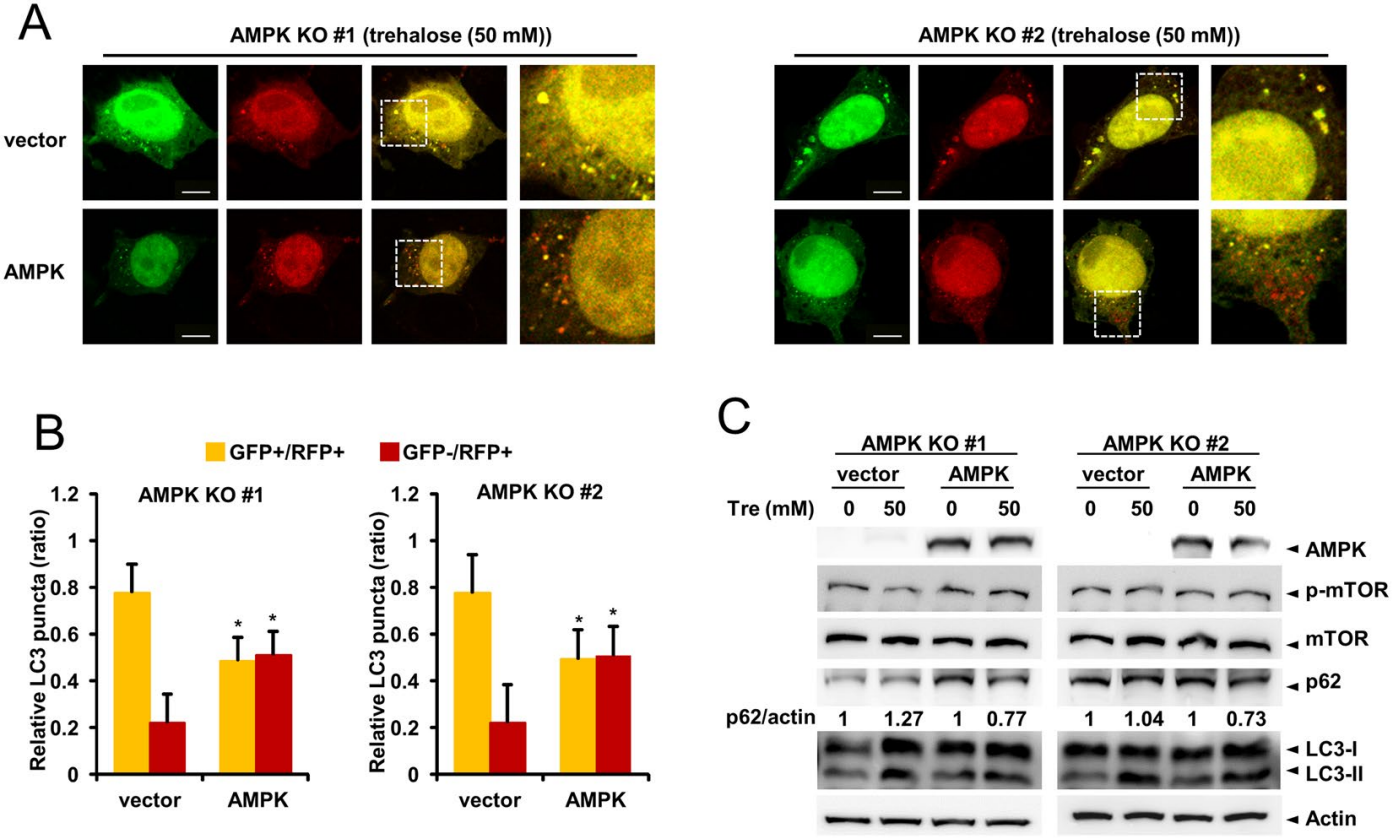
Expression of AMPK α1 recovers trehalose induced autophagic flux. (**A**,**B**) AMPK α1 knockout cells were transfected with mRFP-GFP-LC3 in the presence or absence of the AMPK plasmid. Twenty-four hour after, cells were treated with trehalose for 6 h. Ratio quantification of autophagosomal LC3 (GFP+/RFP+) puncta to autolysosomal LC3 puncta (GFP−/RFP+) Bars: 10 μm. (n = 15, vector vs AMPK plasmid, *P < 0.0001). (**C**) AMPK α1 knockout cells were transfected with AMPK plasmid. Twenty-four hour after transfection, cells were treated with trehalose for 6 h. Cell lysates were probed with the indicated antibody.

To further confirm these results, we examined whether autophagosomes could fuse with lysosomes in AMPK α1 knockout cells upon trehalose treatment. Control cells and AMPK α1 knockout cells were transfected with mRFP-LC3. Twenty-four hours after transfection, the cells were treated with trehalose, followed by immunostaining with anti-LAMP1 antibody. We found that autolysosome formation is impaired in AMPK α1 knockout cells (Fig. 7D,E). Finally, we performed a rescue experiment by expressing AMPK α1 in AMPK α1 knockout cells. The overexpression of AMPK α1 restores the autophagosome maturation by forming autolysosomes (Fig. 8A,B). We also examined p62 levels and show that the restoration of AMPK α1 decreased the level of p62 upon trehalose treatment (Fig. 8C). These results indicate that AMPK α1 knockout does not disrupt the trehalose-induced formation of autophagosomes, but does delay autolysosome formation by inhibiting autophagosome maturation.

## Discussion

Here, we report that AMPK increases autophagic flux by contributing to autophagosome maturation and autolysosome fusion. The role of AMPK in autophagy initiation has been clearly shown in previous studies^12,13^; however, the role of AMPK in the later stages of autophagy, autophagosome maturation, and autolysosome fusion has not been characterized. We showed that AMPK α1 depletion impaired the progression of autophagy induced by compound C treatment. This indicates that AMPK α1 contributes to the progression of autophagy, as well as autophagy initiation.

We generated AMPK α1 knockout cell lines using the CRISPR-Cas9 gene editing method in HEK293T cells. Although there are two isoforms of the AMPK α subunit, AMPK α1 and AMPK α2, the knockout of only AMPK α1 impaired autophagosome formation under starvation in HEK293T cells, suggesting that AMPK α1 plays an important role in starvation-induced autophagy in HEK293T cells (Fig. 2). Since we generated the knockout cells with the HEK293T cell line, in which the transfection efficiency is high, we obtained definitive results using the autophagy marker mRFP-GFP-LC3. In addition, we used two different guide RNAs to avoid biased results due to off-target effects.

It is controversial whether compound C promotes or inhibits autophagy. It has been reported that compound C inhibited autophagy by suppressing AMPK activity in U2OS osteosarcoma cells, while another study reported that compound C induced autophagy in U251 glioma cells by an AMPK-independent pathway^19,27^. In a previous report that claimed that compound C induced autophagy by an AMPK-independent pathway, the authors did not examine the later stages of autophagy, such as autophagosome maturation^19^. Here, we demonstrated that compound C induced autophagosome formation via an AMPK-independent manner. However, cells treated with compound C require AMPK for the maturation of autophagosomes and for autolysosome formation. Therefore, our experimental results provide another explanation for compound C-induced autophagy.

Since compound C inhibits AMPK activity, it is quite paradoxical that AMPK is involved in compound C-induced autophagy by promoting autophagosome maturation. We used a high concentration of compound C (10 μM) and found that the AMPK activity is significantly reduced. Therefore, our results imply that this reduced AMPK activity is sufficient for autophagosome maturation and autolysosome formation.

Because compound C is also an AMPK inhibitor, it is difficult to make a clear conclusion about the role of AMPK α1 in autophagy from these experiments. However, we also showed that autophagosome formation was normal and autolysosome formation was impaired in AMPK α1 knockout cells in the presence of trehalose, an mTOR-independent autophagy inducer. Taking both the trehalose and compound C experiments together, we conclude that AMPK α1 is required for efficient autophagosome and autolysosome formation during autophagy.

Although autophagosome maturation and autolysosome formation are significantly impaired in AMPK α1 knockout cells, we did not find a significant change in cell viability upon treatment with compound C (Fig. 5B,C). These results are consistent with previously published results that apoptosis induced by compound C is AMPK independent^28,29^. In this respect, autophagy induced by compound C is neither cytoprotective nor cytotoxic, but rather nonprotective^4,30^. Compound C has been reported to sensitize cancer cells to drug-mediated apoptosis^31,32^. Therefore, compound C might sensitize tumor cells to other cancer drugs by regulating autophagy.

In this report, we showed that AMPK expression is required for efficient autophagosome maturation and lysosome fusion in AMPK α1 knockout cells. Since we tested our hypothesis in specific conditions and in a specific cell line, the requirement of AMPK for efficient autophagosome maturation and lysosome fusion might not apply to other conditions. Therefore, further experiments will be required to confirm our results in other cell lines and other conditions.

## Materials and Methods

### Cell culture and cell proliferation assay

HEK293T cells were grown in DMEM medium (Welgene, Korea) supplemented with 10% fetal bovine serum (Gibco, Waltham, MA, USA) and 1% antibiotic-antimycotic solution (Welgene, Seoul, Korea). Cell proliferation was measured using the [4,5-dimethylthiazol-2-yl]-2,5-diphenyltrazolium bromide (MTT) assay. Briefly, cells were seeded in a 24-well plate and then incubated overnight. At the indicated time, MTT solution was added to a final concentration of 1 mg/ml and incubated for 3 additional hours. Trypan blue assay was performed to measure cell viability. Cells were stained with trypan blue solution and the numbers of live cells and dead cells were counted by a TC10 automated cell counter (Bio-Rad, Richmond, CA, USA). MTT was purchased from USB Corporation (Cleveland, OH, USA) and Compound C from Sigma (St. Louis, MO, USA). Compound C was dissolved in DMSO (stock solution, 10 mM) and control cells were treated with an equal volume of DMSO (vehicle alone).

### Generation of the AMPK α1 knockout cell line with CRISPR/Cas9

Guide RNA sequences for use in the CRISPR/Cas9 system were designed at the CRISPR design website (http://crispr.mit.edu/), provided by the Feng Zhang Lab^24^. The sequences for the insert oligonucleotides for human AMPK α1 gRNA #1 and #2 were as follows 5′-CACCGA AGATCGGCCACTACATTC-3′/5′-AAACGAATGTAGTGGCCGATCTTC-3′and 5′-CACCGCCGAGAAGCAGAAACACGA-3′/5′-AAACTCGTGTTTCTGCTTCTCGGC-3′, respectively. The AMPK α1 guide RNA targets exon 1 of the AMPK α1 gene. The complementary oligonucleotides for the guide RNAs (gRNAs) were cloned into a pX459 CRISPR/Cas9-Puro vector (Addgene, Cambridge, MA, USA). HEK293T cells were transfected with lipofectamine transfection reagent (Invitrogen, Carlsbad, CA, USA). Two days after transfection, cells were treated with 1 μg/ml of puromycin (Sigma) for three days. After two weeks, colonies were isolated, and the AMPK α1 sequences were amplified by PCR and analyzed using the T7 endonuclease (T7E1) assay, DNA sequencing (Macrogen, Seoul, Korea), and Western blotting. The T7E1 enzyme was purchased from New England Biolabs (Beverly, MA, USA).

### Western blotting

For Western blot analysis, polypeptides in whole cell lysates were resolved by SDS-PAGE and transferred to nitrocellulose membrane filters. Proteins were detected with a 1:1000 or 1:5000 dilution of primary antibody using an enhanced chemiluminescence (ECL) system. Images were acquired using the Chemidoc-it 410 imaging system (UVP, Upland, CA, USA) and LAS4000 system (GE Healthcare, Uppsala, Sweden). The following primary antibodies were used: anti-AMPK α1 (Cell Signaling Technology, Beverly, MA, USA), anti-phospho-AMPK α1 (Cell Signaling Technology), anti-LC3 (MBL international, Watertown, MA, USA), anti-p62 (MBL international**)**, anti-acetyl-CoA carboxylase (ACC) (Cell Signaling Technology), anti-phospho acetyl-CoA carboxylase (p-ACC) (Cell Signaling Technology), and anti-actin (ABM, Richmond, BC, Canada).

### Immunofluorescence and confocal microscopy

HEK293T wild-type and AMPK knockout cells were grown on sterilized glass coverslips. After plasmid transfection, cells were fixed with 4% paraformaldehyde. For immunostaining, cells were blocked with 10% goat serum (Gibco) in phosphate-buffered saline (PBS; Wellgene, Seoul, Korea), stained with a 1:500 dilution of primary antibody in PBS, and then stained with a 1:1000 dilution of fluorescence-conjugated secondary antibody (Invitrogen, Carlsbad, MA, USA). Finally, slides were washed three times with PBS and mounted in mounting medium containing DAPI (Vector Laboratories, Burlingame, CA, USA). Images were captured with a Carl Zeiss LSM710 confocal microscope (Oberkochem, Germany). The plasmid pTF-LC3 was purchased from Addgene (Cambridge, MA, USA) and the anti-LAMP1 antibody from Santa Cruz Biotechnology (Santa Cruz, CA, USA).

### Transmission electron microscopy

HEK293T cells were sequentially fixed with 2.5% glutaraldehyde and 1% osmium tetroxide on ice for 2 hours and washed with PBS. The tissues were then serially dehydrated in ethanol and propylene oxide, embedded in an Epon 812 mixture, and polymerized in an oven at 70 °C for 24 hours. The sections acquired from the polymerized blocks were collected on grids, counterstained with uranyl acetate and lead citrate, and examined with a Bio-HVEM system (JEM-1400Plus at 120 kV and JEM-1000BEF at 1000 kV, JEOL, Tokyo, Japan).

Autophagosome was identified by its contents (morphologically intact organelles) with the existence of double membranes (partly visible), and autolysosome was identified by its contents (partially degraded cellular organelles due to cellular autolysis) with the clear empty space. Lysosome can be identified by its homogenous content.

### Statistical methods

The results of the western blot, TEM data and LC3 puncta analysis were evaluated by a 2-tailed *t* test using Excel software (Microsoft, Seattle, WA, USA). *P* < 0.05 was considered significant.

## Electronic supplementary material

Supplementary Figures

## Data Availability

The datasets generated during and/or analyzed during the current study are available from the corresponding author on reasonable request.
