# Supplementary material for: AMPK contributes to autophagosome maturation and lysosomal fusion
Source: Sci Rep. 2018 Aug 23;8:12637. doi: 10.1038/s41598-018-30977-7 (PMC6107659; doi:10.1038/s41598-018-30977-7)
Supplement: Supplementary file 1 — Supplementary Figures [file 41598_2018_30977_MOESM1_ESM.docx]

Supplementary information for:

**AMPK contributes to autophagosome maturation and lysosomal fusion**

by Minsu Jang, Rackhyun Park, Hyunju Kim, Sim Namkoong, Daum Jo, Yang Hoon Huh, Ik-Soon Jang, Jin I Lee and Junsoo Park


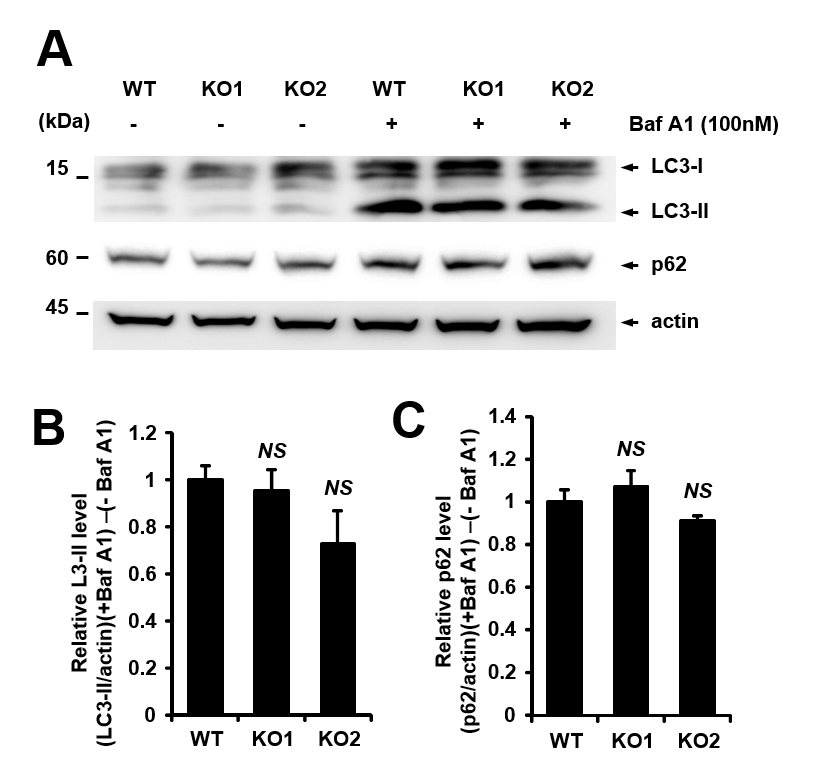


**figure S1**. Bafilomycin A1, a lysosomal inhibitor, blocked the basal autophagic flux in control and AMPK knockout cells. Control cells and AMPK α1 knockout cells were treated with bafilomycin A1 (100 nM) for 4h. We measured the autophagic flux as the amount of LC3-II in the absence of lysosomal inhibitors subtracted from the amount of LC3-II in the presence of lysosomal inhibitors for each of the conditions: (+ Baf A1) – (– Baf A1). We calculated the level of p62 in the same manner. Control vs knockout cells, NS: not significant.

The cropped blots are used in this figure and full-length blots are presented in supplementary figure S12.


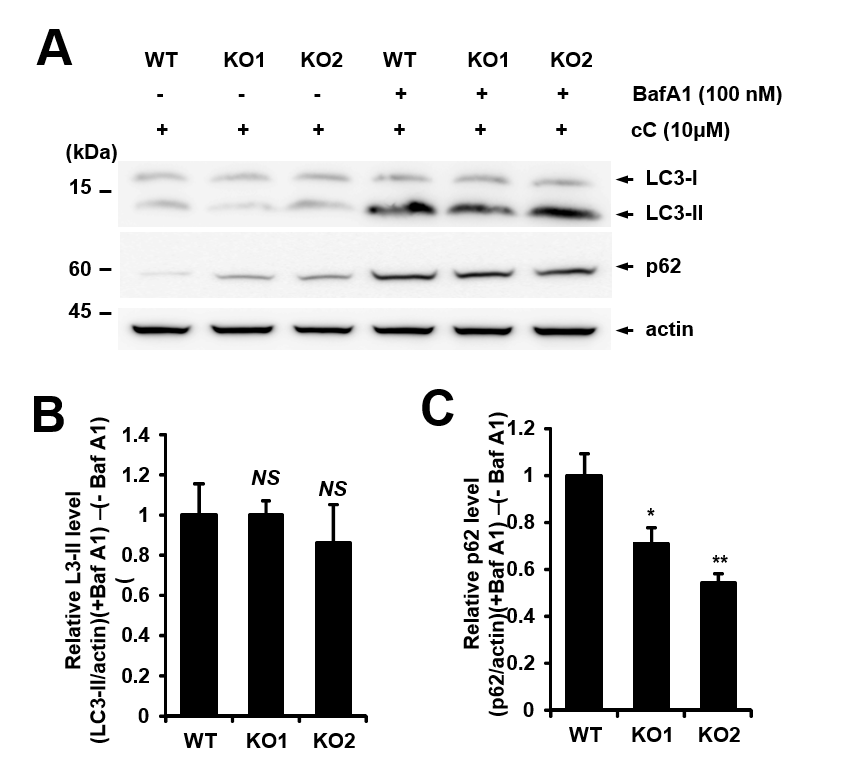


**figure S2**. AMPK knockout cells showed decreased autophagic flux upon compound C treatment. Bafilomycin A1 was used to block compound C induced autophagic flux in control and AMPK knockout cells. Control cells and AMPK α1 knockout cells were treated with compound C in the presence or absence of bafilomycin A1 (100 nM) for 4h. We measured the autophagic flux as the amount of LC3-II in the absence of lysosomal inhibitors subtracted from the amount of LC3-II in the presence of lysosomal inhibitors for each of the conditions: (+ Baf A1) – (– Baf A1). We calculated the level of p62 in the same manner. Control vs knockout cells, *: P <0.05, **: P<0.005. The cropped blots are used in this figure and full-length blots are presented in supplementary figure S13.

**
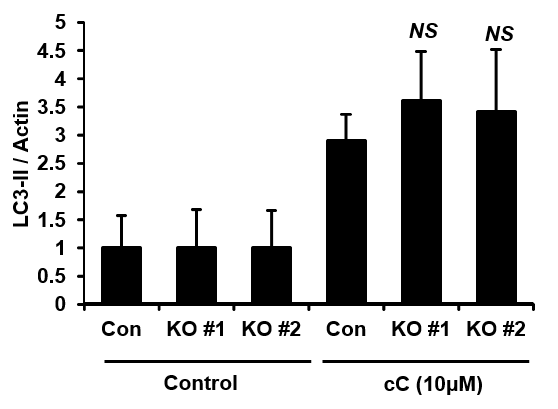
**

**figure S3**. AMPK knockout did not affect the level of LC3-II. HEK293T cells and AMPK α1 knockout cells were treated with compound C (10 μM) for 18 h, and the cell lysates were probed with anti-LC3 and anti-actin antibodies. The LC3-II bands were quantified (n=4), and the relative expression levels are shown in the graph. Control cells vs. AMPK α1 knockout cells, NS: not significant.


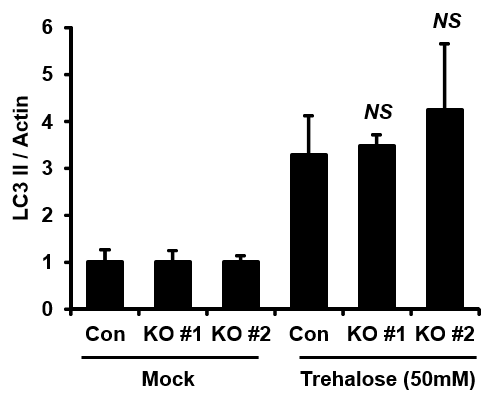


**figure S4**. AMPK knockout did not affect the level of LC3-II. HEK293T cells and AMPK α1 knockout cells were treated with trehalose (50mM) for 6 h, and the cell lysates were probed with anti-LC3 and anti-actin antibodies. The LC3-II bands were quantified (n=3), and the relative expression levels are shown in the graph. Control cells vs. AMPK α1 knockout cells, NS: not significant.


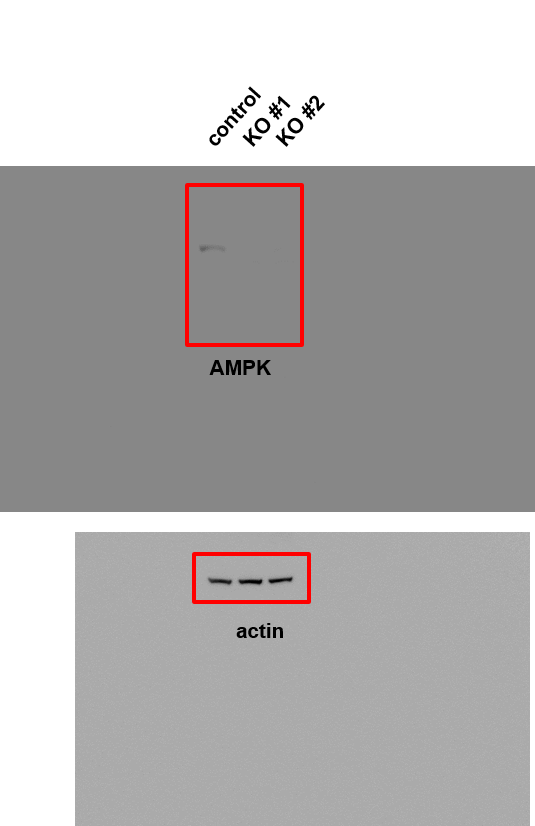


**figure S5**. The full-length blots of figure 1C.


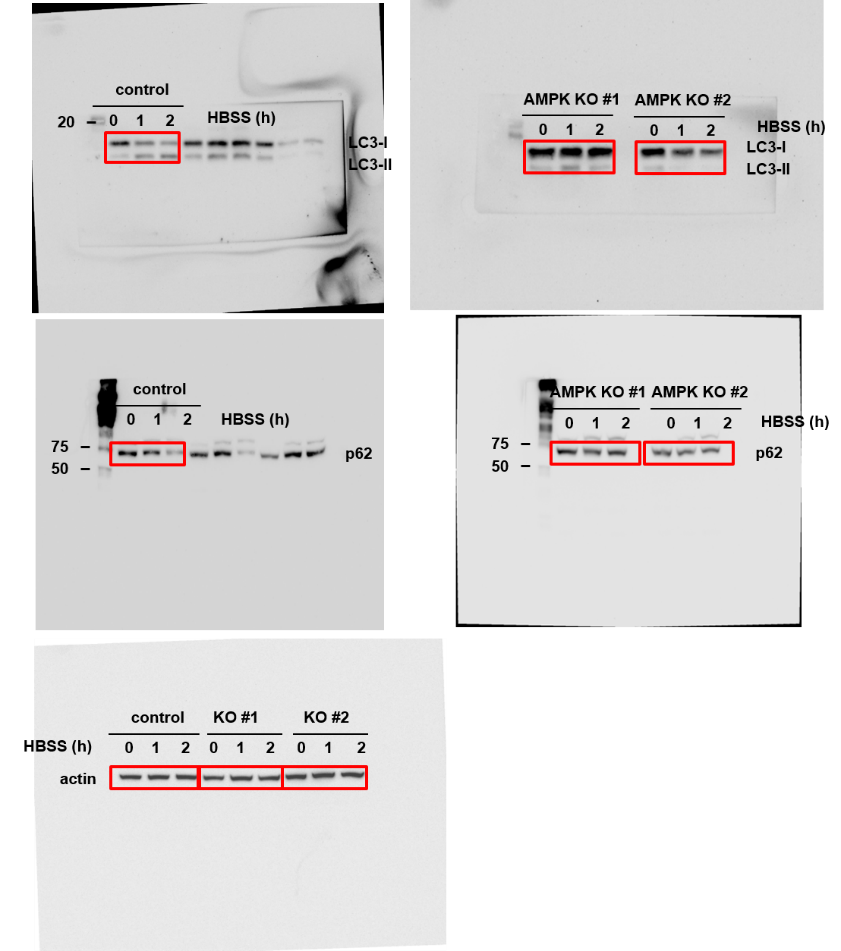


**figure S6**. The full-length blots of figure 2C.


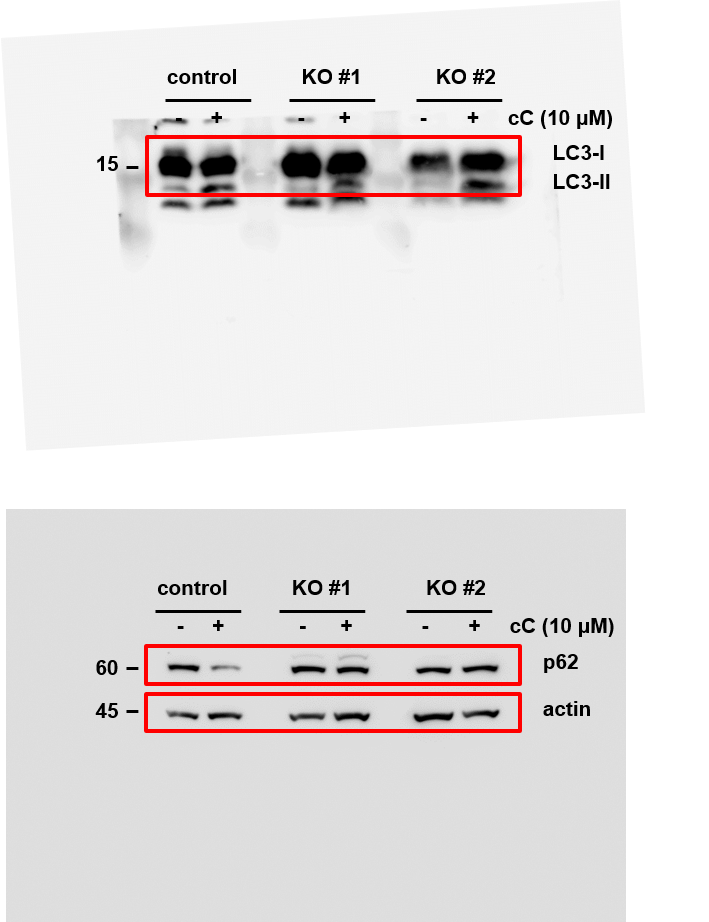


**figure S7**. The full-length blots of figure 3C.


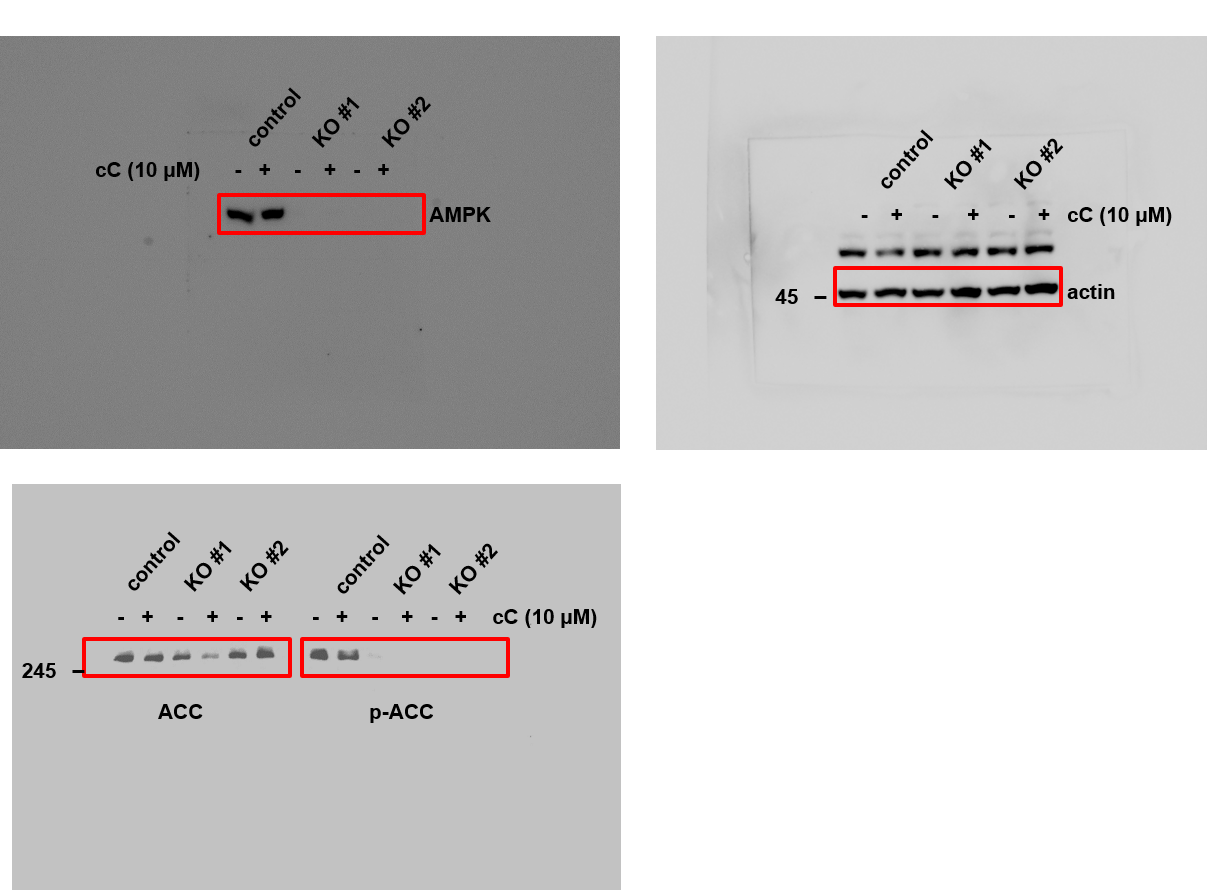


**figure S8**. The full-length blots of figure 5A.


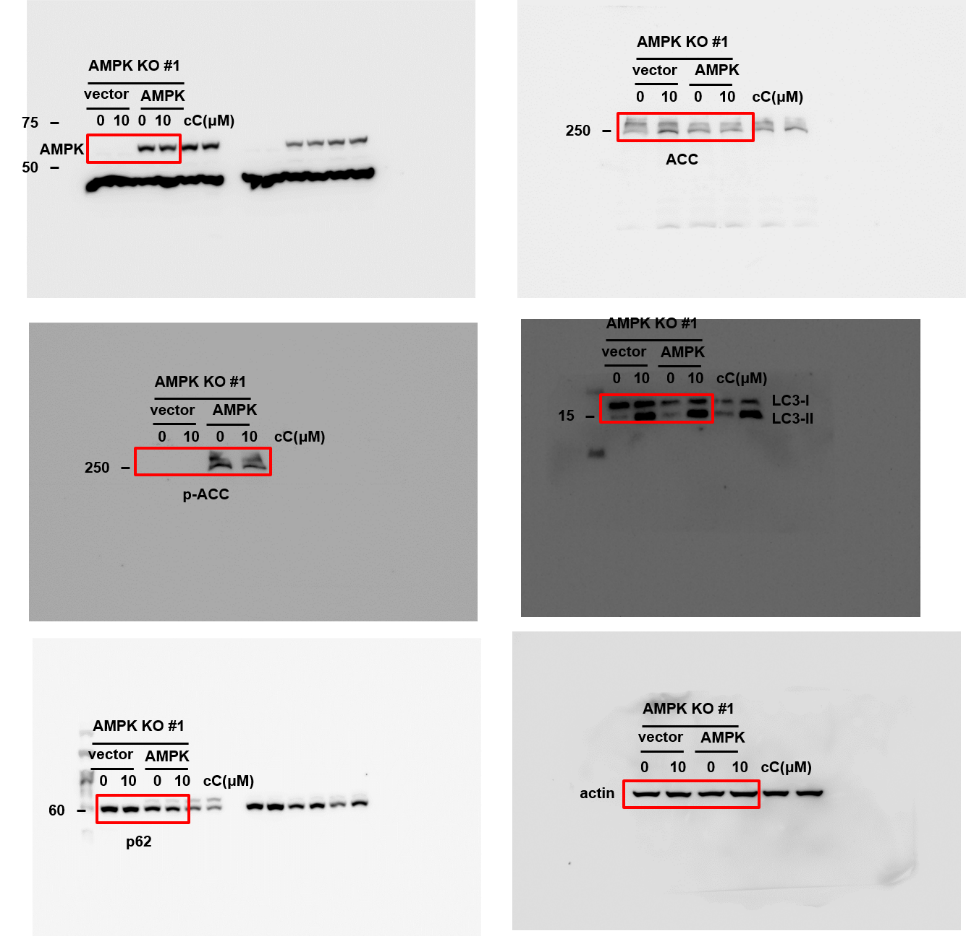


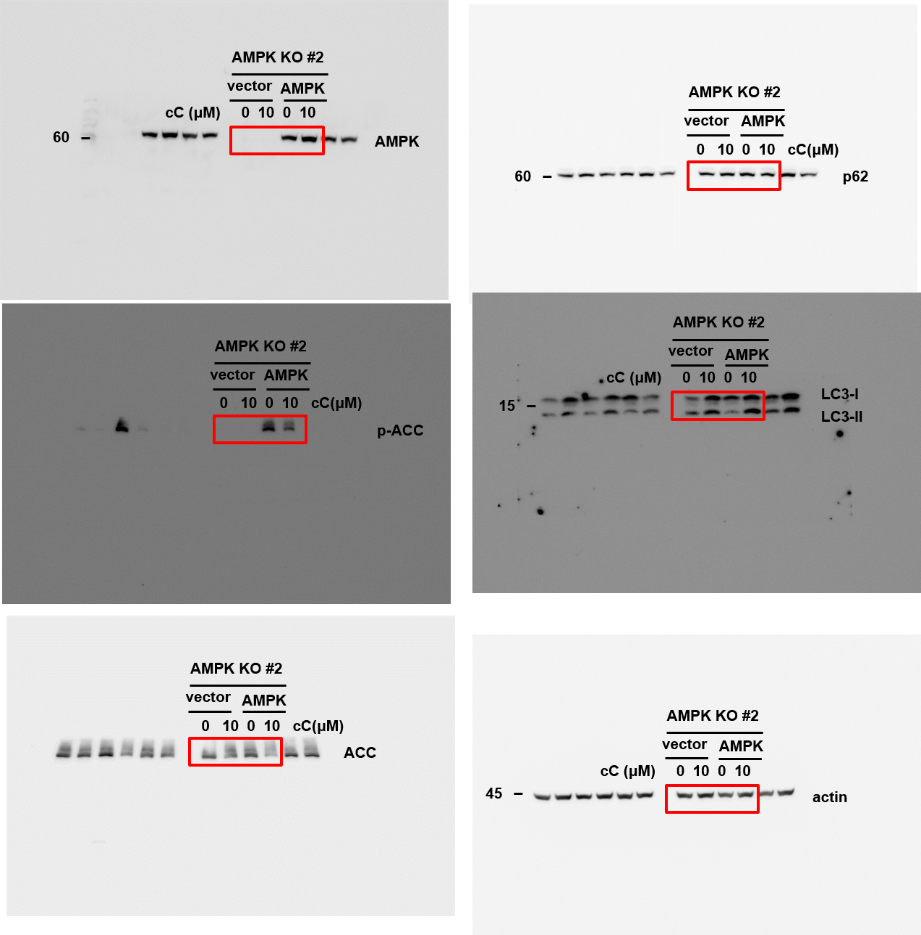


**figure S9**. The full-length blots of figure 6C.


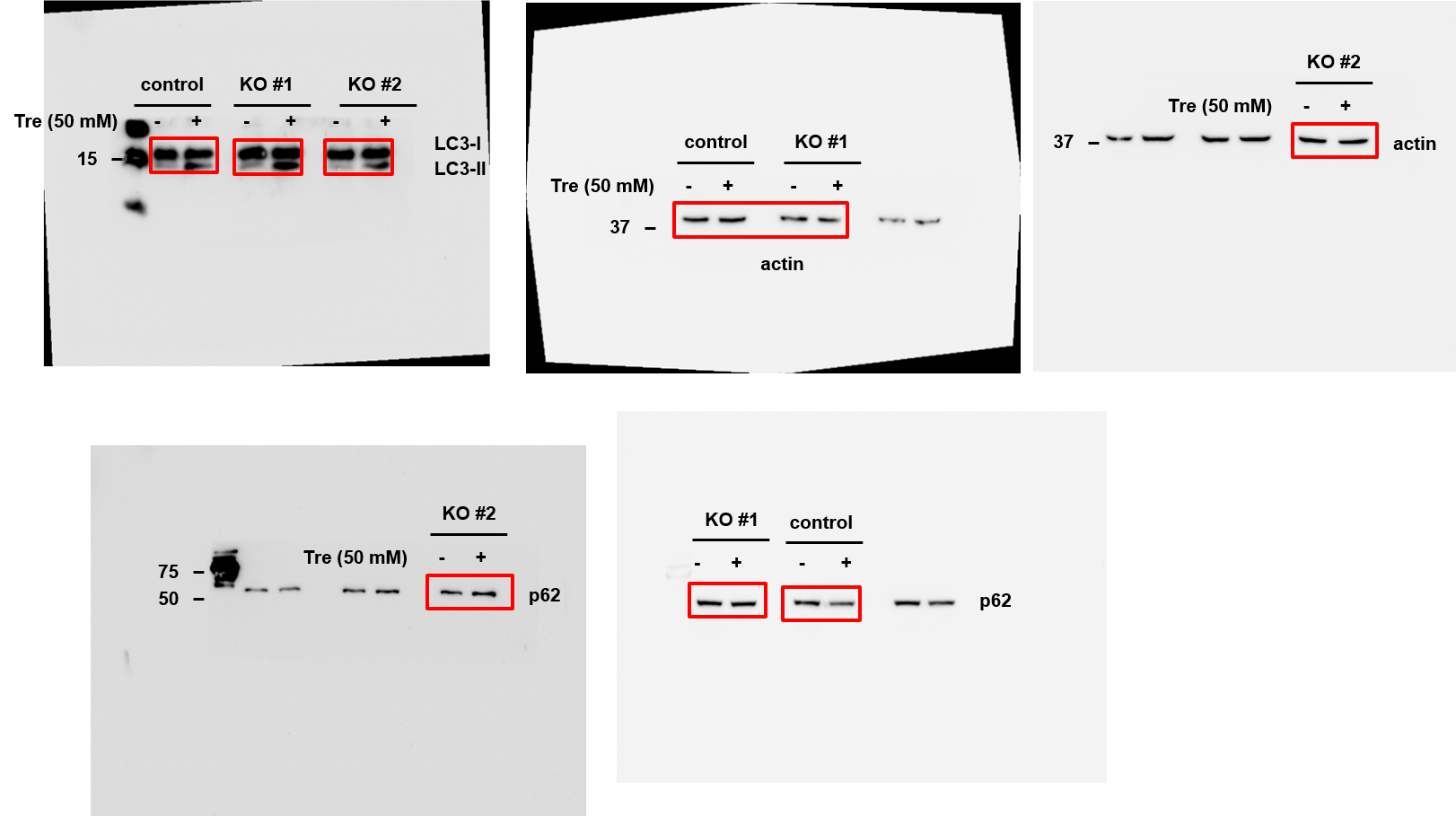


**figure S10**. The full-length blots of figure 7B.


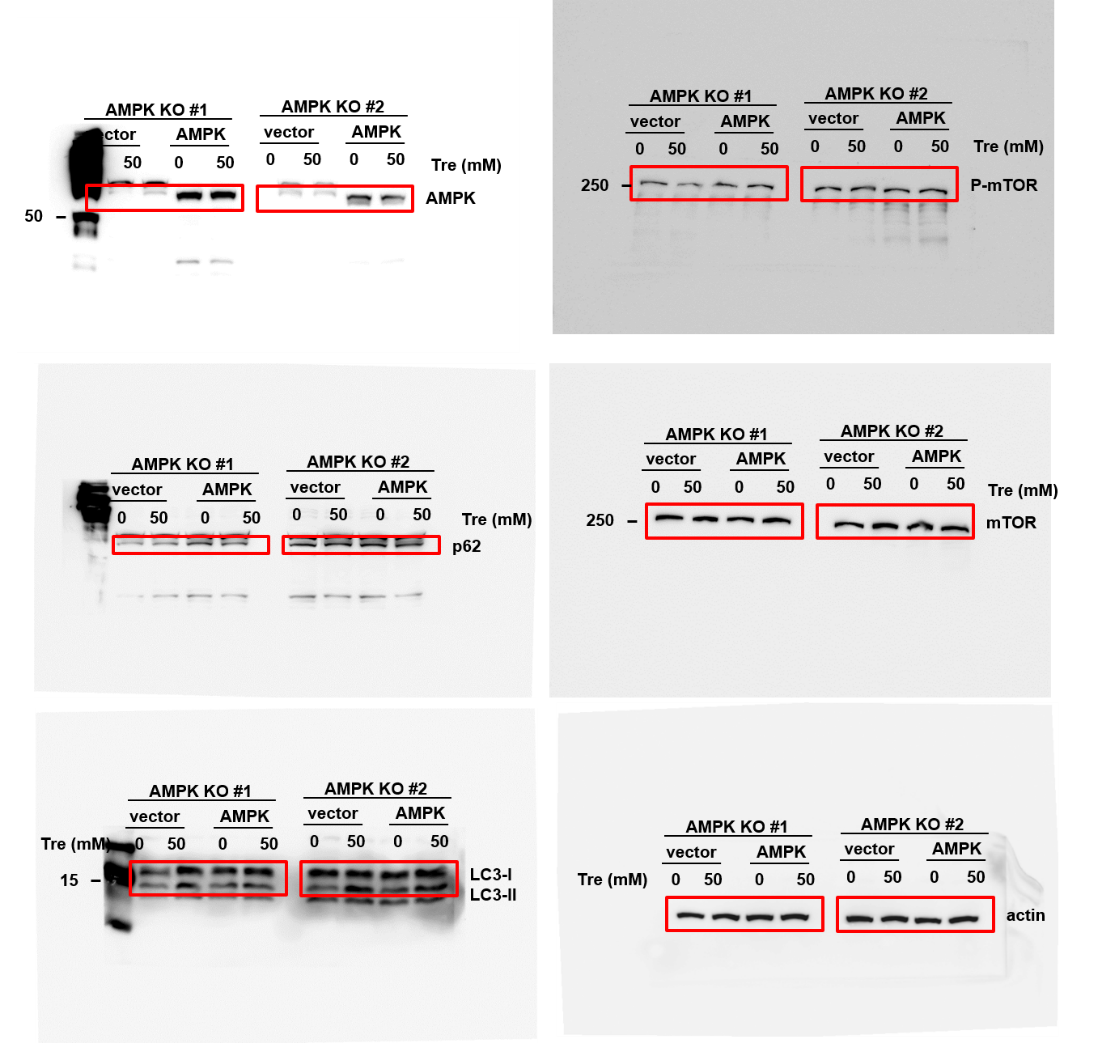


**figure S11**. The full-length blots of figure 8C.


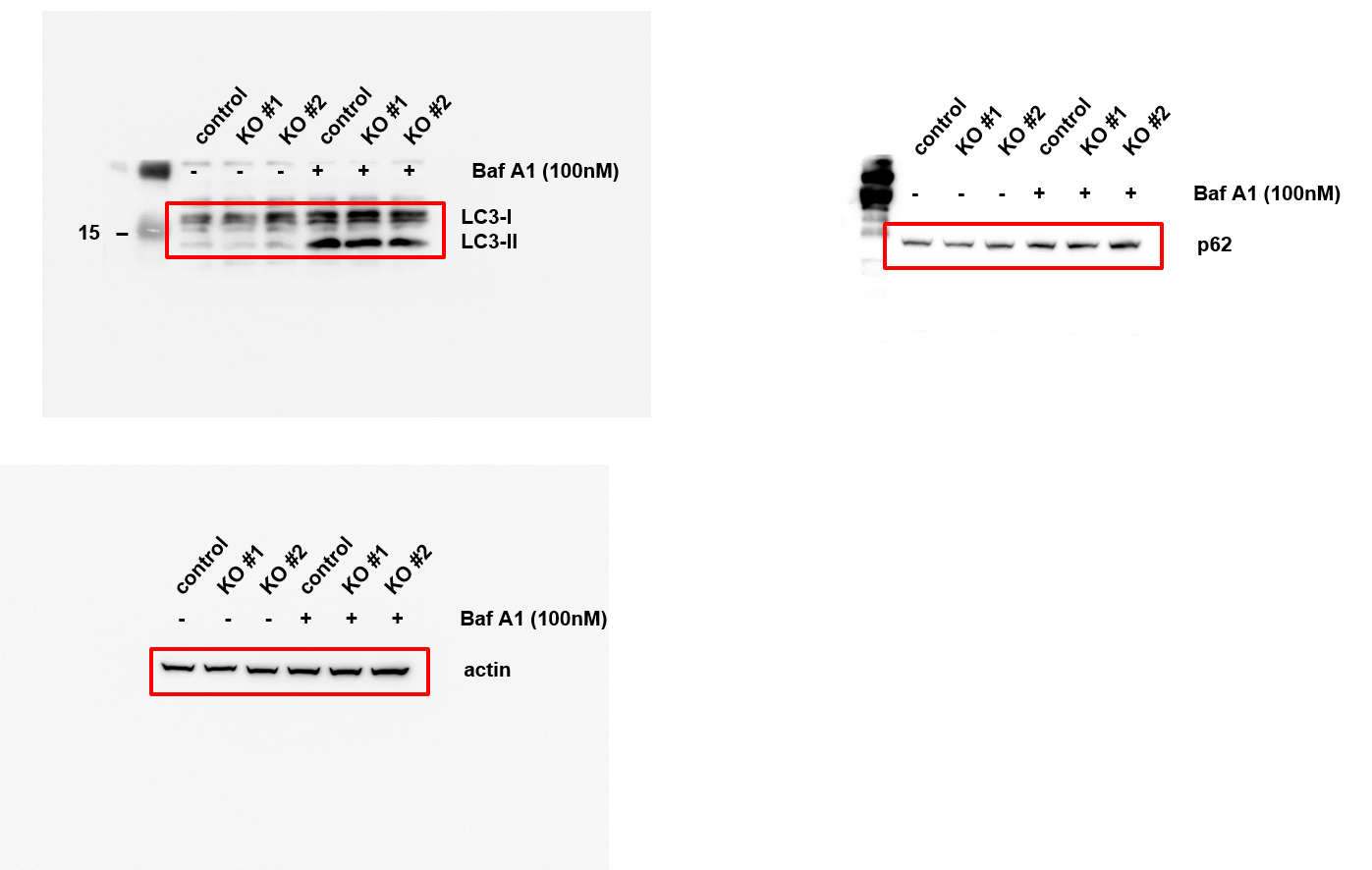


**figure S12**. The full-length blots of figure S1.


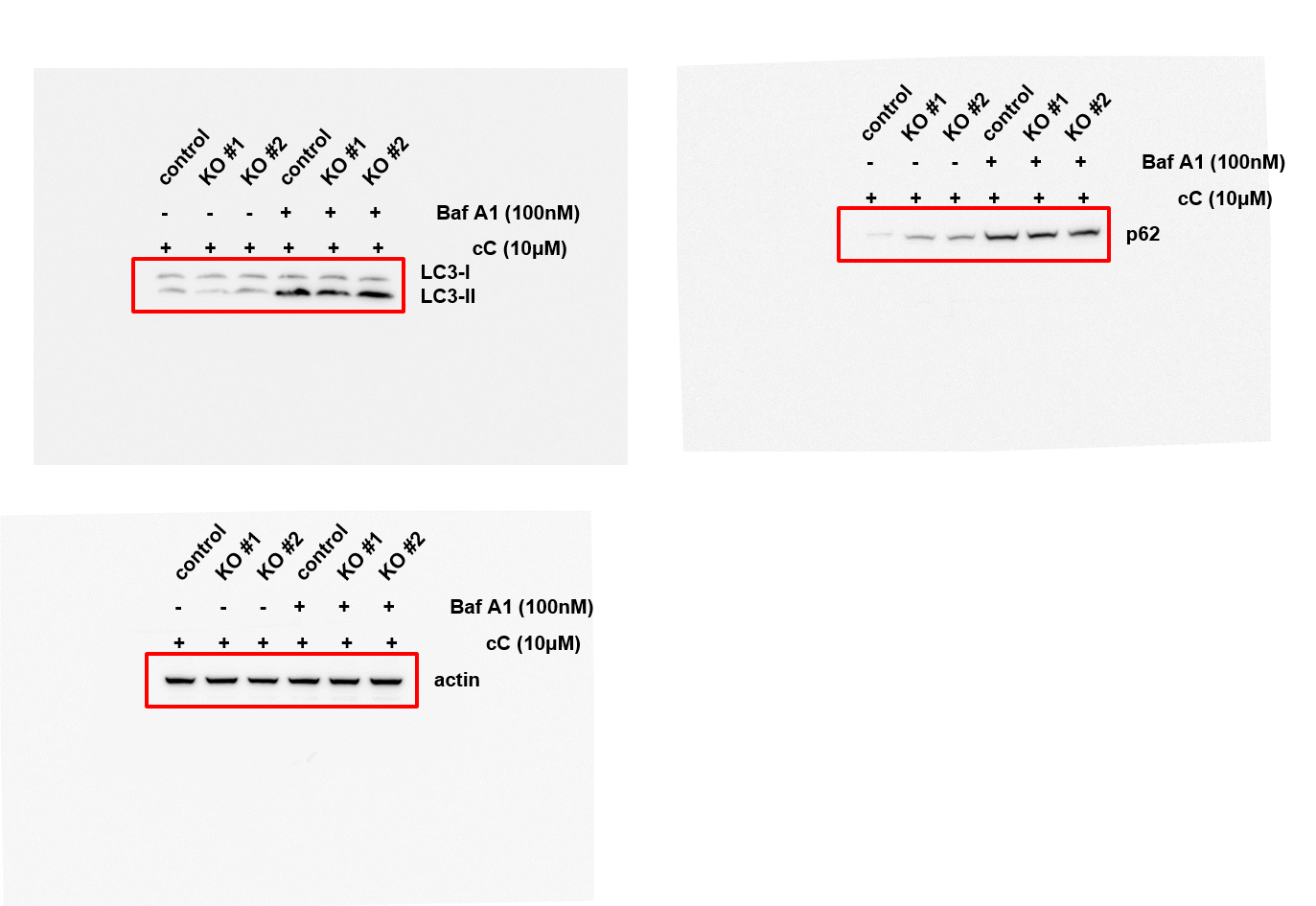


**figure S13**. The full-length blots of figure S2.
